# Contribution of Liver Nonparenchymal Cells to Hepatic Fibrosis: Interactions with the Local Microenvironment

**DOI:** 10.1155/2017/6824762

**Published:** 2017-02-16

**Authors:** Michel Fausther, Michele T. Pritchard, Yury V. Popov, Kim Bridle

**Affiliations:** ^1^Division of Gastroenterology and Hepatology, Department of Internal Medicine, University of Arkansas for Medical Sciences, Little Rock, AR 72205, USA; ^2^Department of Pharmacology, Toxicology and Therapeutics, University of Kansas Medical Center, Kansas City, KS 66160, USA; ^3^Division of Gastroenterology and Hepatology, Beth Israel Deaconess Medical Center, Harvard Medical School, Boston, MA 02215, USA; ^4^Liver Research Unit, Gallipoli Medical Research Institute, University of Queensland, Greenslopes, QLD 4120, Australia

Hepatic fibrosis is a hallmark feature of chronic liver diseases of various etiologies including genetic deficiency, metabolic derangement, infection, autoimmunity, and exposure to physical and chemical agents. This condition yearly affects millions of patients in the USA and across the world. Progressive hepatic fibrosis, leading to cirrhosis, is the most common cause of liver failure.

Essentially, hepatic fibrosis represents a pathophysiological process triggered in response to liver injury, to mediate tissue repair and wound healing. It is primarily driven by specialized effector cells called liver myofibroblasts and characterized by sustained extracellular matrix deposition, leading to excessive scar formation, organ dysfunction, and eventually end-stage liver disease cirrhosis. As fibrosis takes place, persistent cell damage and distinctive changes in liver tissue architecture trigger in parenchymal and nonparenchymal hepatic cells several signal transduction pathways that regulate pathophysiological mechanisms promoting tissue repair, such as inflammation, proliferation, remodeling, and angiogenesis. Concomitantly, the newly formed extracellular matrix scaffold evolves into a dynamic and complex microenvironment that orchestrates, with damage-activated hepatic cells such as leukocytes and liver myofibroblasts, key functional aspects of the response to liver injury such as regulated release of fibrogenic/proliferative factors and cell activation/migration, to restore tissue homeostasis and integrity. Yet, in spite of significant advances in the identification of key molecular targets and signaling pathways, there is presently no effective and well-tolerated curative strategy for hepatic fibrosis in patients, beyond liver transplantation.

This special issue focuses on current concepts relevant to the pathogenesis of hepatic fibrosis. We hope that these articles will generate continued interest in understanding of the molecular mechanisms driving hepatic fibrosis progression.

Three articles will focus on how molecular changes of the homeostatic status of extracellular matrix (ECM) network, including ECM transcriptome alterations (L.G. Poole and G. E. Arteel), ECM-modifying protein activities (T. Saneyasu et al.), or ECM remodeling regulation (C. L. Lamb et al.), significantly impact the progression of hepatic fibrosis.

The extracellular matrix (ECM) is more than an inert scaffold on which epithelial cells are anchored and through/over which cells travel. It is increasingly appreciated that the ECM is a dynamic entity in all tissues and serves as a critical depot for growth factor sequestration required for rapid response to tissue injury or other cues. In addition, degradation of ECM components themselves leads to the liberation of bioactive fragments that modulate response to tissue injury. Critically, direct interactions between cells and the ECM, mediated predominantly by integrins, induce potent signals, which dictate cell activity such as migration. These, and other examples, demonstrate the critical importance of the ECM in regulation of tissue homeostasis in healthy tissue and in return to homeostasis after tissue injury. When dysregulated, as in the case of chronic liver injury, ECM remodeling favors the accumulation of ECM components. In liver disease, much effort is placed on understanding the pathomechanisms that drive fibrosis, focusing on development of myofibroblasts, synthesis of fibrillar collagens, and scar “maturation.” This strategy is employed to help in the development of bona fide antifibrosis therapies. While it is indisputable that a fibrotic ECM is responsible for organ dysfunction in advanced liver disease, early, “transitional” changes to the ECM likely drive fibrogenesis. Understanding these transitional changes may reveal novel points of intervention to prevent the development of liver fibrosis in the first place. In their contribution to this special issue, L. G. Poole and G. E. Arteel provide strong evidence to support this idea. Specifically, several changes in the ECM and associated molecules, referred to as the “matrisome,” are known in alcoholic liver disease and these changes occur prior to histologic evidence of fibrosis. Further discussed are how the current approaches to studying the ECM are flawed as they often consider single ECM components in isolation and at the expense of understanding the myriad of other ECM changes which happen concurrently. This reductionist approach fails to fully appreciate the incredible complexity associated with a full understanding of matrisome-modifying events in early liver disease. To fill this important gap, an interesting proposition is to utilize a robust “omics” approach to more thoroughly understand transitional ECM changes associated with early stages of liver disease progression, a novel and intriguing idea.

Once the ECM proteins are secreted, enzymatic cross-linking of the major ECM proteins (collagens, elastin, and others) is an essential process for fibrotic matrix stabilization, which in turn contributes to fibrosis progression and limits reversibility of liver fibrosis once the causative agent is removed. Moreover, collagen cross-linking confers increased stiffness to fibrotic matrix, promoting activation of fibrogenic effector cells through mechanosensing. As T. Saneyasu et al. review in this issue, several enzymes, including tissue transglutaminase (TG2) and the lysyl oxidase (LOX) family, are overexpressed in hepatic fibrosis and able to catalyze the formation of collagen cross-links. However, TG2-deficient mice display unaltered collagen cross-linking, develop liver fibrosis normally, and do not show improved fibrosis reversal, casting doubt on the functional significance of TG2 in fibrotic matrix stabilization. Instead, lysyl oxidases directly participate in fibrotic matrix cross-linking and stabilization, since LOX inhibition favorably affects the architecture of liver scar tissue, rendering fibrosis more readily reversible after cessation of the fibrogenic stimulus. In the liver, hepatic stellate cells and portal fibroblasts are major producers of LOX family proteins, including LOXL2 that has recently been implicated to promote lung and liver fibrosis and tumorigenesis. Anti-LOXL2 therapeutic antibody (simtuzumab) is currently undergoing testing in phase II clinical studies in patients with primary sclerosing cholangitis, nonalcoholic steatohepatitis, and hepatitis C/HIV coinfection (NCT identifier: 01672866, 01672879, 01672853, and 01707472), while several small molecule LOX/LOXL2 inhibitors are currently in early stages (preclinical/Phase 1) of development.

There is a notable paucity of studies examining the effect of environmental/nutritional toxins or xenobiotics (e.g., smoke toxicants, drugs) on hepatic fibrosis, a dysregulated tissue repair process associated with the development of chronic liver conditions. Because xenobiotics often tend to be considered as disease-enhancing rather than disease-causing agents, their regulatory role in hepatic fibrosis has been underappreciated to an extent. However, that view has slowly evolved with the advent of functional studies directly linking the biological activities of xenobiotic-sensing nuclear receptors to alterations of liver homeostasis including organ toxicity, tissue damage, and tissue repair. Recently, environmental pollutant 2,3,7,8-tetrachlorodibenzo-p-dioxin (TCDD), a xenobiotic acting as ligand for aryl hydrocarbon receptor, has been shown to exacerbate development of hepatic fibrosis; however the mechanisms by which this occurs have not been fully explored. The study by C. L. Lamb et al. in this issue examined the impact of TCDD-induced activation of aryl hydrocarbon receptor on the regulation of ECM synthesis, deposition, and breakdown during chronic liver injury. The authors described three components of ECM homeostasis, namely, collagen synthesis, ECM metabolism, and plasminogen activator/plasmin system, that were regulated by TCDD. Further insights into the mechanisms responsible for this dysregulation will be important contributions to our understanding of ECM homeostasis in hepatic fibrosis following exposure to environmental pollutants.

The next three articles will highlight the complexity of functional cooperation between various specialized liver cell populations and its critical importance in hepatic fibrosis progression, in the context of inherited liver disease (L. Jiang et al.), metabolic dysfunction (N. Magee et al.) specifically, or chronic liver diseases (V. Natarajan et al.) in general.

In addition to chronic overnutrition and hepatotoxins (e.g., alcohol exposure), genetic diseases exist in which hepatic fibrosis is a major contributor to disease morbidity and mortality. One such disease is congenital hepatic fibrosis found in patients with autosomal recessive polycystic kidney disease (CHF/ARPKD). Disease pathogenesis is linked to mutations in a protein called fibrocystin, which normally localizes to primary cilia found on cholangiocytes and renal tubule epithelial cells. Those mutations lead to a lack of fibrocystin localization to primary cilia and malformed primary cilia, which suffer functional defects leading to the development of large, fluid-filled cysts and a robust pericystic fibrosis. However, a clear understanding of the mechanisms driving CHF/ARPKD progression remains elusive in this understudied and rare disease. In this issue, L. Jiang et al. propose a “pathogenic triumvirate” to explain CHF/ARPKD progression and link cell proliferation and cyst growth and fibrosis as interrelated pathomechanisms. Indeed, several identifiable commonalities between molecular drivers of proliferation, inflammation, and fibrosis in CHF/ARPKD disease exist in support of this notion and may altogether indicate that the mechanistic relationships between the major cell types found in each of the triumvirate's vertices including myofibroblasts, macrophages, mast cells, and proliferating cyst wall epithelial cells that produce and transduce those shared molecular signals are the integral mediators of disease progression. In this context, these proven commonalities shared between pathomechanisms could be either targeted independently, to break the feed-forward cycle of progressive disease, or targeted by way of combinatorial therapies aimed at each of the vertices, to further enhance disease regression. Finally, and perhaps even more intriguing, it is conceivable that each member of the triumvirate is regulated by the same core mechanism, thus raising the possibility that a single target for therapeutic development may exist. Overall the review stresses the importance of cell-cell and cell-matrix cross talk in mechanisms which drive fibrotic disease, emphasizing the importance of nonreductionist approaches to studying disease pathogenesis and progression.

Nonalcoholic fatty liver disease (NAFLD) is a common liver disorder that describes a wide range of chronic liver conditions caused by steatosis, a process histologically characterized by abnormal lipid accumulation within hepatocytes. In a small group of patients, it can progress to a potentially serious condition called nonalcoholic steatohepatitis (NASH), which occurs when lipid deposition is combined with sustained inflammation, severe cell damage, and advanced fibrosis ultimately leading to cirrhosis. Currently, effective therapeutic options are not available, partly because understanding of NASH pathogenesis remains limited. From the initial “two-hit hypothesis” proposed by Day and Jamesto to the current “multiple parallel hits hypothesis” articulated by Tilg and Moschen, the prevailing line of thought has been that NASH represents a multifaceted disorder that does not mechanistically revolve around steatosis development solely. Conceptually, an important question that has not yet been conclusively answered is whether steatosis development precedes, coincides with, or even follows NASH development. As N. Magee et al. emphasize in this special issue, NASH is increasingly recognized as a complex interplay between parenchymal cells and nonparenchymal cells. Early research efforts have mainly focused on elucidating the genetic factors and molecular signals associated with lipid toxicity, oxidative stress, and organelle dysfunction (e.g., endoplasmic reticulum, mitochondria) in parenchymal hepatocytes that were initially seen as the primary source of pathogenic factors driving NAFLD/NASH development. More recently, novel signaling pathways relevant to disease pathogenesis have been uncovered in nonparenchymal cell populations including immune cells and resident liver fibroblasts that play critical roles in inflammation initiation/perpetuation and scar tissue formation process associated with NAFLD progression to NASH, respectively. Innate/acquired immunity mechanisms such as cytokine production/release, inflammasome activation, and gut dysbiosis are being recognized as important factors that contribute to prolonging hepatic steatosis and exacerbating inflammation in NASH. In addition, major signaling molecules such as Notch and sonic Hedgehog that promote fibrosis progression in NASH have been now identified as key regulators of activation of hepatic stellate cells, a fibroblast population from which most of injury-associated myofibroblasts originate in several chronic liver disease settings. These recent developments shed a new light on key molecular pathways underlying NAFLD/NASH pathogenesis and will lead to the identification of novel therapeutic targets and strategies for NASH condition.

Liver sinusoidal endothelial cells (LSEC) have recently emerged as an important regulator of both progression and regression of liver fibrosis. Liver endothelium is positioned at the functional interface, receiving molecular cues from ECM, nonparenchymal cells, hepatocytes, and blood, as reviewed in depth by V. Natarajan et al. in this issue. In the normal liver, quiescent fenestrated LSEC suppress activation of hepatic stellate cells, losing such ability when liver vasculature undergoes “capillarization” in chronically injured liver. A recent study demonstrated that manipulation of specific pathways selectively in LSEC might force the liver to either regenerative (via chemokine receptor CXCR7) or fibrotic responses (via chemokine receptor CXCR4 and growth factor receptor FGFR1) to chronic liver injury, thereby postulating the fundamental importance of liver vasculature in chronic liver disease outcomes. Also, neovascularization (angiogenesis) has been noted in chronic liver disease for decades, although its important (and complex) role in liver fibrosis has not been appreciated until recently. During progressive phase of chronic liver injury, suppression of angiogenesis may impact liver fibrogenesis differently depending on particular pathway. Thus, vascular endothelial growth factor (VEGF) neutralization suppresses angiogenesis and fibrosis in mice, but inhibition of neovascularization via integrin *α*v*β*3 antagonism may worsen fibrotic outcomes. Contrarily, during recovery phase, VEGF is required for liver repair and fibrosis resolution, reminiscent of dual, opposing role of Cd11b+ macrophages during injury and repair.

Another article in this special issue will describe the regulation of liver myofibroblast functions by fibroblast growth factor-associated signaling pathways and its functional relevance in hepatic fibrosis progression.

In chronic liver diseases, liver myofibroblasts drive the wound healing and tissue repair response, by releasing large amounts of extracellular matrix (e.g., collagens, glycosaminoglycans). Several studies have shown that the perennial cellular source of liver myofibroblasts during fibrosis is the nonparenchymal hepatic stellate cell population. Following injury, hepatic stellate cells (HSC) undergo several phenotypic changes including heightened contractility, increased fibrogenic capacity, and enhanced sensitivity to inflammatory, fibrogenic, and proliferative mediators. Among the latter, the fibroblast growth factor (FGF) signaling pathway has emerged as an important regulator of HSC-derived liver myofibroblast functions. As J. D. Schumacher and G. L. Guo review in this issue, various FGFs (FGF1, FGF2, FGF7, FGF9, FGF15/FGF19, and FGF21 among others) and their related receptors (FGFR1–4) are expressed by HSC-derived liver myofibroblasts, in regulated fashion during the development of liver fibrosis. Also, FGF molecules induce multiple signal transduction cascades within liver myofibroblasts, to control key cellular functions/processes such as activation state, proliferative ability, and migratory capacity. Despite potential functional redundancy resulting from pharmacological promiscuity (e.g., receptor-ligand binding affinity), the central role played by FGFs and FGFRs activities in the regulation of liver myofibroblasts functions indicates that these molecules may represent attractive therapeutic candidates for the development of antifibrotic treatments.

The liver is a functionally complex organ, owing to both its cellular diversity and its specialization. Hepatic fibrosis, commonly defined as excessive deposition of ECM proteins in the local microenvironment, is the scar forming process triggered in response to chronic liver injury. Altogether, the articles included in this special issue underlines the importance of liver functional complexity during the development of hepatic fibrosis but also stresses the significance of dynamic interactions existing between both cellular (parenchymal and nonparenchymal cells) and noncellular (ECM proteins, ECM-associated proteins, and ECM-modifying proteins) components of the hepatic microenvironment during hepatic fibrosis. A better understanding of the molecular mechanisms underlying these cell-to-cell and cell-to-matrix interactions will give new insights into the pathogenesis of liver fibrosis.

*Michel Fausther* *Michele T. Pritchard* *Yury V. Popov* *Kim Bridle*

